# *Drosophila melanogaster* as a neurobehavioral model for sex differences in stress response

**DOI:** 10.3389/fnbeh.2025.1581763

**Published:** 2025-07-11

**Authors:** Elisabeth Tawa, Daniel Notterman

**Affiliations:** ^1^Princeton Neuroscience Institute, Princeton University, Princeton, NJ, United States; ^2^Department of Molecular Biology, Princeton University, Princeton, NJ, United States

**Keywords:** stress, *Drosophila*, sex differences, social isolation, cellular and molecular

## Abstract

Sex differences are observed in several neurologic and psychiatric disorders. Many aberrant behavioral symptoms can be characterized clinically as either internalizing or externalizing, which tend to manifest disproportionately in females or males, respectively. Stress may precipitate or amplify these behavioral disturbances, which often start in childhood and adolescence but persist into adulthood. Increased understanding of sex differences in stress-induced behavioral changes and their underlying molecular mechanisms is integral to developing better therapeutics specifically tailored to males and females. Here, we highlight the potential of *Drosophila melanogaster* (*D. melanogaster*) as a model for the neurobiological study of sex differences in stress-altered behavior. We first review paradigms for stressing *D. melanogaster*, with an emphasis on social environmental stress. We then introduce behavioral tests that can be used to quantify stress-induced behaviors in flies and note sex differences that emerge in response to stress. Finally, we provide an overview of the known molecular and cellular mechanisms underlying stress-induced behavioral change, with a focus on sex differences and studies incorporating social isolation or crowding.

## Introduction

Across human neuropsychiatric disorders, sex biases manifest through disease prevalence, severity, and development. Late-onset schizophrenia, anorexia, bulimia, post-traumatic stress disorder, anxiety, and depression are more prevalent in female patients, whereas attention deficit hyperactivity disorder, autism, dyslexia, stuttering, Tourette’s syndrome, and early-onset schizophrenia are more frequent in male patients (McCarthy, 2016). Over the life course, women are roughly twice as likely to develop depression and anxiety disorders compared to men (Kessler et al., 1994; Gater et al., 1998; Kuehner, 2017). Although it is important to consider the role of sex in the etiology of human mental illness and the biases that influence diagnosis of certain behavioral disorders, establishing relationships between biological sex and aberrations in behavior is the first step to elucidating those behaviors’ neurobiological underpinnings.

### Sex differences in human disorders

One lens through which we can consider the contribution of biological sex to the development of maladaptive behavior is that of internalizing and externalizing psychopathology. Psychologists have long established internalizing and externalizing as broad categories of behaviors in humans, particularly in children and adolescents (Achenbach, 1966). Internalizing behavior is thought to be self-focused, while externalizing behavior relates to one’s interaction with the social environment. Internalizing behaviors include behaviors suggesting anxiety, depression, and emotional dysregulation. Externalizing behaviors encompass episodes of aggression, impulsivity, deviance, and hyperactivity (Nikstat and Riemann, 2020). One recent behavioral genetic study using data from over 3,000 twin families attributed roughly one-third of variance in internalizing and externalizing behavior to genetic influences, leaving the majority of variance to be accounted for by environmental factors, such as stress (Nikstat and Riemann, 2020). The authors suggest that many previous heritability estimates for internalizing and externalizing behaviors may be inflated, reflective of the “missing heritability” problem (Maher, 2008), whereby heritability estimates differ based on whether they were calculated using twin data or DNA-based approaches such as genome-wide association studies (GWAS).

Furthermore, there are prominent sex differences in the prevalence of internalizing versus externalizing disorders in human children and adolescents. Sexual selection theory posits that differences between boys and girls in biomarkers, molecular mechanisms, and development can produce differences in risk and resilience for various forms of psychopathology (Martel, 2013). Boys may be more predisposed to externalizing risk factors such as disinhibition and sensation-seeking due to higher levels of prenatal testosterone exposure, which result from the male fetus’ production of hormones in the testes beginning around week 8 of gestation (Wilson et al., 1981). This enhanced prenatal testosterone is also hypothesized to make males more susceptible to prenatal stressors (Martel and Roberts, 2014; Barrett and Swan, 2015) with downstream effects on dopaminergic signaling in the brain (Martel, 2013). Alternately, girls may be more at risk for developing internalizing disorders, with the risk markers being negative affect and rumination. These risk factors may arise during puberty from increases in estradiol and its interaction with cortisol and oxytocin, which may act on the serotonergic system to increase females’ stress sensitivity (Goel and Bale, 2010; Martel, 2013). Taken together, in human children and adolescents, psychologists posit that stress exposure in conjunction with differences in sex hormones may trigger different monoaminergic signaling processes in male versus female brains, which manifest as sex-specific stress phenotypes.

### Comparative utility of model systems in the study of stress response

While mammalian models have proven useful for gaining mechanistic understanding of how stress differentially impacts male versus female brains and translationally relevant behaviors, *Drosophila melanogaster* (*D. melanogaster*) offers certain advantages. Beyond the decreased costs and ethical considerations of working with an invertebrate species, flies have a shorter lifespan than rodents, thus facilitating the study of stress and its effects at different developmental timepoints. In *D. melanogaster*, it is also simple to manipulate the sex determination hierarchy in certain cellular populations without manipulating the sex phenotype of the entire organism or its hormones. This strategy can be harnessed to perform sex-reversal studies, which shed light on how the genetic sex of a small neuronal population can regulate behaviors (Mundiyanapurath et al., 2009; Oyeyinka et al., 2022). The ease of sex determination hierarchy manipulation in combination with the recent mapping of the *D. melanogaster* connectome (Winding et al., 2023) positions *D. melanogaster* as a promising model system for identifying sex-specific neural circuits with fine-scale resolution.

While these qualities have made *Caenorhabditis elegans* (*C. elegans*) a good model system for studying the interrelationships among molecular mechanisms, neural circuit function, and behaviors that differ by sex (Salzberg et al., 2021), *D. melanogaster* enables us to study more complex behaviors with excellent availability of genetic tools and greater conservation of genes relevant to human disease (Sonnhammer and Durbin, 1997; Lloyd and Taylor, 2010). The simple body plan and rapid reproduction of *C. elegans* make it useful for studying cell-to-cell interactions or performing high-throughput genetic analyses (Riddle et al., 1997). However, with a more complex body plan (Alberts et al., 2002), higher genetic homology to humans, and superior availability of genetic tools (Irion and Nüsslein-Volhard, 2022), *D. melanogaster* may be a more useful invertebrate system for modeling human disease. Although flies lack a vertebrate nervous system, the complexities of mammalian hormonal signaling via sex hormones, and morphological and anatomical similarities to humans, fundamental molecular pathways have been found to be highly conserved across species in multiple neurological diseases (Rubin and Lewis, 2000). *D. melanogaster* has been used to discover new molecular pathways and interactions in transgenic fly models of neurodegenerative and metabolic brain diseases, epilepsy, tumors, and neurotrauma (Jeibmann and Paulus, 2009).

### Overview

The goal of this review is to present *D. melanogaster* as a simple yet elegant model system through which we can probe the molecular mechanisms of sex-specific behavioral changes following stress experience. We begin by highlighting approaches that have been developed for stressing flies in the laboratory, with a focus on social isolation and crowding stress. We next summarize behavioral tests and key molecules that experimenters have studied in flies to assess phenotypes such as motivation, locomotion, aggression, activity, and sleep. Then, we consider sex differences in how these behaviors change in response to social or physical stressors. We conclude with an extensive discussion of the molecular mechanisms and neuromodulatory systems underlying *D. melanogaster* stress-induced behavioral changes, with an emphasis on the effects of social isolation and crowding and how they may differ between males and females.

### *Drosophila melanogaster* as a model system for studying stress

A vast literature exists on stress and its phenotypic and behavioral effects in male and female *D. melanogaster* (Table 1). Numerous paradigms have been developed to subject *D. melanogaster* to physical or psychosocial stress. Physical stressors include starvation (Neckameyer and Weinstein, 2005), oxidative stress-inducing chemical exposure (Neckameyer and Weinstein, 2005; Rzezniczak et al., 2011), temperature (Yang et al., 2013), mechanical stress (Neckameyer and Weinstein, 2005; Ries et al., 2017), sleep deprivation (Huber et al., 2004; Tanizawa and Takemoto, 2021), and electric shocks (Batsching et al., 2016). Social stressors consist of modifications to the fly’s social environment, including social isolation (Brown et al., 2017; Yadav et al., 2024) and overcrowding (Sørensen and Loeschcke, 2001; Lall et al., 2019; Chen and Sokolowski, 2022). Even the presence of dead conspecifics has been shown to impact fruit flies adversely through changes to the head metabolome and decreases in longevity (Chakraborty et al., 2019).

**Table 1:** Known stress-induced phenotypes and behavioral effects in *D. melanogaster*.

Form of stress	Sex	Phenotypes	References
Chronic unpredictable mild stress (heat, cold, starvation, sleep deprivation)	Males	Aggression ↑; depression-like behavior ↑ (forced swim test and light–dark box test); body weight ↓; preference for sucrose ↓	Araujo et al. (2018)
Social isolation	Males & females	Sleep ↓; daytime activity ↑	Ganguly-Fitzgerald et al. (2006)
	Males	Sleep ↓; daytime activity ↑	Agrawal et al. (2019)
	Males	Sleep ↓; activity ↑; feeding ↑	Li et al. (2021)
	Males	Aggression ↑	Wang et al. (2008) and Agrawal et al. (2020)
	Females	Aggression ↑	Ueda and Kidokoro (2002)
Adult crowding	Males & females	Longevity ↓	Joshi and Mueller (1997)
	Males	Sweet food-seeking ↑; activity ↑	Lall et al. (2019)
	Females	Sweet food-seeking ↓; activity ↓; exploration ↓	
Chronic mechanical stress (vibration)	Males	Sweet food-seeking ↓; activity ↑	Lall et al. (2019)
	Females	Sweet food-seeking ↓; activity ↑; exploration ↓	
Mechanical stress (vibration) + food and water deprivation	Males	Depression-like behavior ↑ (motivation to climb)	Ries et al. (2017)
Short-term variable stress (social isolation, fasting, heat, electric shock)	Males & females	Preference for sucrose ↓; activity ↑	Ramos-Hryb et al. (2021)
Starvation	Females	Activity ↑	Yu et al., 2016
	Males	Aggression ↑	Edmunds et al. (2021)
Lifelong same-sex pairing	Males	Longevity ↓; climbing ability ↓	Leech et al. (2017)
	Females	Longevity ↓; climbing ability ↑	

Some models of *D. melanogaster* stress use a chronic unpredictable mild stress approach, in which multiple stressors are used in combination (Ries et al., 2017; Araujo et al., 2018, 2020). For example, flies may be exposed to heat, cold, starvation, and sleep deprivation at unpredictable intervals over a period of 10 days (Araujo et al., 2018, 2020). Chronic unpredictable mild stress in male *D. melanogaster* has been shown to induce both aggression and depression-like behavior, measured through significantly greater numbers of aggressive encounters between male pairs and faster times to immobility in a forced swim test relative to controls (Araujo et al., 2018). The same study also found chronic unpredictable mild stress approach to decrease body weight, decrease preference for sucrose, reduce percentage of time spent in the illuminated compartment of a light–dark box (the preferred side of the apparatus for flies), and diminish whole head levels of serotonin and dopamine (Araujo et al., 2018). Although these tests imply depressive-like, anhedonia-like, and defensive behaviors in flies following chronic stress, it is important to consider ethological relevance in adapting animal behavior tests traditionally developed for rodents to flies. For instance, interpreting a reduction in time spent in the light portion of a light–dark box as a readout of depressive or defensive behavior is plausible in flies, given that flies demonstrate positive phototaxis and have been found to employ anti-predation strategies (Ferreira and Moita, 2020). However, while rodents generally avoid bright light as prey animals, young adult flies are attracted to light. These interspecies differences must be accounted for when modifying rodent behavioral tests for flies, and not all tests may be adaptable. For example, the forced swim test might not be easily translatable from rodents to flies. The interpretation of this test has not only been debated in the rodent literature (Molendijk and de Kloet, 2022), but rodents are largely considered natural swimmers, whereas flies are not. Thus, swimming might not be a behavior that makes sense to study in *D. melanogaster* in the laboratory, and an interpretation of the fly immobility response as behavioral despair is likely over-anthropomorphizing.

### Social isolation

In studies of the effects of an individual stressor, social isolation has largely been used in *D. melanogaster* to examine the behavioral impact of environmental deprivation. Although fruit flies are not eusocial insects and thus are not commonly viewed as a model for sociality, they can demonstrate behaviors reflective of affective states (Anderson and Adolphs, 2014) and social learning (Sokolowski, 2010). *D. melanogaster* flies can form social networks (Schneider et al., 2012; Pasquaretta et al., 2016; Jezovit et al., 2021), perform collective behaviors (Wu et al., 2003; Durisko et al., 2014; Ramdya et al., 2017), and transmit mating preferences (Mery et al., 2009; Danchin et al., 2018). Laboratory assays have been developed for quantifying social space (Simon et al., 2012) and social attraction (Sun et al., 2020) within groups of flies, and freely-moving flies of both sexes demonstrate attraction to immobilized flies, regardless of sex, in a shallow circular chamber (Sun et al., 2020). In the absence of social interaction, flies show alterations in their lifespan (Leech et al., 2017; Lin et al., 2022; Vora et al., 2022), gene expression in the brain (Agrawal et al., 2020; Li et al., 2021), and behaviors (Wang et al., 2008; Li et al., 2021; Vora et al., 2022). Social interaction, relative to isolation, in male *D. melanogaster* has been found to modulate an array of behaviors, including aggression, feeding, and sleep (Ganguly-Fitzgerald et al., 2006; Chen and Sokolowski, 2022). These behavioral changes can persist for up to 72 h following social experience and correlate with increases in CREB-dependent neural activity and synaptic plasticity in the mushroom bodies (Gil-Martí et al., 2024). Another report suggests that social experience may drive social motivation in *D. melanogaster* through serotonergic signaling and activation of neurons in the *γ* lobe of the mushroom body (Sun et al., 2020). Hence, social interactions may be important for maintaining normal fly physiology, and disruption to sociality in the form of social isolation may induce stress and stress-associated physiological changes.

The social environment has been found to alter *D. melanogaster* sleep patterns such that socially isolated flies sleep less during the day compared to group-housed controls (Ganguly-Fitzgerald et al., 2006). Transcriptional changes in dopaminergic neurons specifically may be responsible for isolation-induced disruptions to sleep (Agrawal et al., 2019). A more recent study of social isolation in *D. melanogaster* replicated and extended these findings to a feeding context by establishing mechanistic connections between social deprivation, activity, and metabolism. This group found that chronic social isolation reduces sleep, enhances feeding behavior, and induces transcriptomic changes indicative of a starvation-like brain state in male flies (Li et al., 2021). Aggression is another behavior that increases in laboratory flies following social isolation (Hoffmann, 1987; Wang et al., 2008; Agrawal et al., 2020). This enhancement in aggression occurs in both sexes, with males fighting for food, territory, and mates (Hoffmann and Cacoyianni, 1989; Zwarts et al., 2012) and females fighting for food and egg-laying sites (Ueda and Kidokoro, 2002; Nilsen et al., 2004). Downregulation of the neuropeptide drosulfakinin (Dsk), a fly ortholog to mammalian cholecystokinin (CCK), has been identified as the potential neural substrate of chronic social isolation-induced aggression (Agrawal et al., 2020).

Finally, social experience and isolation have been shown to regulate male courtship maturation in *D. melanogaster*. Group-housing, compared to social isolation, increases mature males’ courtship behavior via enhanced activity of olfactory receptor neurons expressing the Or47b pheromone receptor (Sethi et al., 2019). Downstream signaling cascades convey information about reproductive maturity and population density, which can be integrated to modulate courtship. This specific process, whereby the fly flexibly tunes its reproductive behavior based on a combination of internal and external states, may be coordinated at the molecular level by the male *fruitless* (*fru^M^*) promoter (Zhao et al., 2020). Fru^M^ is a male-specific protein that acts as a master regulator of male courtship behavior. Group housing and signaling via juvenile hormone, a key hormone in the regulation of reproduction and metamorphosis, were found to increase *fru^M^* expression in Or47b neurons and active chromatin marks at the *fru^M^* promoter. However, social isolation or decreased juvenile hormone signaling decreased *fru^M^* expression and enhanced repressive marks around the *fru^M^* promoter.

### Crowding stress

Crowding, the opposite of social isolation, is another potential source of social environmental stress. Much of the existing literature on overcrowding in flies has focused on the effects of larval crowding (Chen and Sokolowski, 2022). In adult flies, this manipulation has ranged from increasing the density of flies within a given physical space to full restraint, imitating chronic restraint stress in rodent models. One study, which employed full restraint, immobilized male flies between two soft plugs for 10 h a day for up to 3 days. These flies were then mated in order to examine the epigenetic, transcriptomic, and metabolic effects of paternal “psychological” stress on the offspring. It was found that paternal restraint stress induced epigenetic changes leading to energy metabolism deficiencies in F1 generation males (Seong et al., 2020). Three days of more moderate adult crowding has also been shown to decrease longevity in adult *D. melanogaster*, though it may be the case that exposure to high density living can build resilience (Joshi and Mueller, 1997).

A study investigating the effects of mechanical stress and adult crowding on behavior also found sex differences in sweet-seeking, activity, and exploration that were more pronounced following crowding than following mechanical perturbation (Lall et al., 2019). Specifically, males demonstrated increased sweet-seeking and activity following adult crowding, whereas females demonstrated reduced sweet-seeking and activity. Females also showed reduced exploratory behavior after chronic crowding. Further research is needed to gain a fuller understanding of the molecular and behavioral impacts of adult crowding in *D. melanogaster*. However, these preliminary findings suggest that crowding could be used in the future as a valid paradigm for uncovering the cellular and molecular mechanisms that generate stress-related behaviors.

### Models for internalizing-like behaviors

Human internalizing disorders are frequently marked by symptoms indicative of depression and anxiety. Although we do not believe flies to possess internalizing emotions like humans, they may experience persistent internal states, which generate behaviors with some resemblance to human internalizing symptoms. In humans, major depressive disorder has been characterized by depressed mood, or bias toward negative emotions; anhedonia, or impaired reward sensitivity; learning and memory dysfunction; appetite and weight changes; and disturbances in sleep and circadian rhythms, among other psychopathological endophenotypes (Hasler et al., 2004). Behavioral despair and anhedonia-like behavior are often interpreted in animal studies as indicative of a depression-like state, as human major depression may be marked by low motivation and persistence, social withdrawal, and a loss of interest or pleasure in enjoyable activities. While animal models cannot capture the full complexity of human depression and internalizing symptoms, they can be useful to the extent that they demonstrate face validity (common behaviors and symptoms), construct validity (common underlying biological mechanisms), and predictive validity (treatment response) (Willner, 1984). Table 2 presents a summary of rodent and fly behavioral tests across various domains of behavior, which reflect a degree of similarity between species.

**Table 2:** Assays used to assess common rodent and fly behaviors.

Behavior	Rodent tests	*D. melanogaster* tests
Depressive-like	Forced swim test (Porsolt et al., 1978; Porsolt et al., 1977) Tail suspension test (Cryan et al., 2005; Can et al., 2012)	Forced swim test (Araujo et al., 2018; Hibicke and Nichols, 2022) Failure to climb (Ries et al., 2017)
Reward/feeding	Sucrose preference (Liu et al., 2018) Sucrose self-administration (Alsiö et al., 2011; Figlewicz et al., 2011) Intracranial self-stimulation (Slattery et al., 2007)	Stop-for-sweet paradigm (Ries et al., 2017; Lall et al., 2019) Sucrose preference (Ramos-Hryb et al., 2021) Capillary action feeding (CAFE) assay (Ja et al., 2007; Diegelmann et al., 2017)
Defensive/anxiety-like	Open field test (Prut and Belzung, 2003; Seibenhener and Wooten, 2015) Light–dark box (Birkett et al., 2011; Kulesskaya and Voikar, 2014) Elevated maze (plus, zero, or T) (Viana et al., 1994; Bertoglio and Carobrez, 2002; Garcia et al., 2011; Tucker and McCabe, 2017) Novelty-suppressed feeding (Bodnoff et al., 1988)	Open field test (Mohammad et al., 2016) Light–dark box (Araujo et al., 2018)
Aggression	Resident-intruder test (Koolhaas et al., 2013)	Aggression assays (Chen et al., 2002; Dierick, 2007)
Sociability	Three-chamber social test (Nadler et al., 2004; Yang et al., 2011) Social interaction test (File and Hyde, 1978; Kraeuter et al., 2019)	Social space assay (vertical triangle test chamber) (Simon et al., 2012) Social approach assay (shallow circular chamber) (Sun et al., 2020)
Locomotor activity	Open field test (Seibenhener and Wooten, 2015)	Open field test (Soibam et al., 2012; Long et al., 2023; Lueningschroer-Wang et al., 2025) Drosophila Activity Monitor (DAM) (Chiu et al., 2010; Pfeiffenberger et al., 2010; Lall et al., 2019)

Common rodent tests of depression-like behavior include the forced swim test and tail suspension test, in which a shorter time to immobility is interpreted as an expression of behavioral despair or entrapment (Lucki et al., 2001; Cryan et al., 2005). Rodent models can be used to study anhedonia-like behavior by measuring a decreased drive for food, sex, or other forms of pleasure through tests such as the sucrose preference test (Liu et al., 2018) or intracranial self-stimulation (Slattery et al., 2007). Similarly, behavioral tests, which will be detailed below, have been developed for flies to quantify stress-induced behavioral despair or anhedonia-like behavior.

### *Drosophila melanogaster* tests of motivation

An important study of fly behavior consistent with learned helplessness found that following exposure to uncontrollable heat pulses, both male and female flies decreased their activity relative to controls and flies that could stop the onset of heat by moving (Yang et al., 2013). Of the flies that endured uncontrollable heat stress, females showed more pronounced reductions in walking and walking speed than males relative to other experimental groups of the same sex. In male flies, it has also been shown that chronic, uncontrollable vibration in combination with food and water deprivation is sufficient to produce what could be interpreted as learned helplessness and anhedonia-like phenotypes (Ries et al., 2017). These behaviors were characterized in male *D. melanogaster* by the failure to climb or the failure of hungry flies to feed on sweet-tasting glycerol, respectively. Upon feeding lithium chloride to male flies, simulating lithium as a pharmacological treatment for major depressive disorder, researchers observed an alleviation of these phenotypes, such that both stressed and control males demonstrated excessive climbing behavior. Serotonergic neurons were found to signal to the fly mushroom bodies to modulate the depression-like state (Ries et al., 2017).

In another study, short-term variable stress consisting of a random sequence of stressors such as social isolation, fasting, heat, and electric shock over a 24 h period was shown to decrease preference for sucrose in male and female flies. However, this phenotype was resistant to pharmacological treatment with fluoxetine and diazepam (Ramos-Hryb et al., 2021). These results are likely due to a combination of stress model design and differing pharmacodynamics between invertebrates and mammals. Because the short-term variable stress protocol encompasses four different types of stressors, it is not clear which of the stressors or which combination of stressors is the key driver of reduced sucrose preference, or how all of these stressors together are impacting the underlying neurobiology. Although the authors seemed to succeed in establishing a stress phenotype prior to treatment administration and behavioral testing, the combination of different stressors may be altering neural signaling in such a way that the primary changes are not serotonergic or GABAergic in nature. Alternatively, it may be that fluoxetine and diazepam differ in their binding affinity to target receptors in flies compared to vertebrate or mammalian models, or that the dose or time of administration was insufficient to ameliorate the stress-induced behavioral change.

Over time, the behavioral neuroscience field has shifted from using animals to model specific mental disorders to using them to understand neurobiological mechanisms underlying general behaviors. In mice and rats, learning how bottom-up neuroendocrine signaling combines with top-down circuit regulation from the medial prefrontal cortex may be key to understanding how animals switch between active and passive coping strategies in the presence of uncontrollable stress (Molendijk and de Kloet, 2022). Functionally analogous neural circuits or gene pathways in flies could be studied in the context of behavioral despair to evaluate whether common mechanisms produce internalizing-like behaviors across species.

### *Drosophila melanogaster* tests of defensive behavior

*Drosophila melanogaster* has also been proposed as a possible model for probing the genetic basis of anxiety disorders. The elevated plus maze, open field, and light–dark box tests have been routinely used in rodents to measure approach or avoidance as a readout of defensive behavior (Griebel and Holmes, 2013). These tests are based on the hypothesis that a more “anxious” animal is thought to avoid light and exposure and prefer darkness and edges, tendencies which are adaptive in the wild for hiding from predators. The tendency of both flies and rodents to avoid the center of the open field arena is called thigmotaxis, centrophobism, or wall-following (WAFO).

In mammals, serotonin has long been the primary neurotransmitter associated with anxiety (Zangrossi et al., 2020), and diazepam is a benzodiazepine medication that is sometimes used in humans for the management of anxiety disorders. Diazepam exerts its anxiolytic effects via allosteric binding at gamma-aminobutyric acid (GABA)-A receptors, which induces neuronal hyperpolarization within the limbic system (Dhaliwal et al., 2023). *D. melanogaster* has orthologous genes which code for the serotonin receptor (1A, 1B, 2A, 2B, and 7), serotonin transporter, and GABA-A receptor. Researchers who measured avoidance behavior in male flies found that pharmacologic administration of diazepam, genetic overexpression of the serotonin class 1 receptor, or overexpression of the serotonin transporter were sufficient to decrease fly WAFO in the open field, similarly to mice (Mohammad et al., 2016). Knock-down of these genes increased mouse and fly WAFO. Some scientists characterize the *D. melanogaster* WAFO behavior as a preference for an arena’s boundaries rather than the centrophobism that mice demonstrate in the open field test (Soibam et al., 2012). However, these authors attributed the arena-boundary preference to the fly’s efficient search for escape routes, and they observed a preference for dark corners in males which may be indicative of shelter-seeking (Soibam et al., 2012). Regardless of whether the preference for spatial boundaries, attraction to the touch of the wall, or fear of the arena center is driving WAFO, these explanations are all rooted in predatory avoidance. A more recent study of WAFO further supports this phenotype as an indicator of emotion-like state in flies, by finding that negatively valenced stimuli (social isolation, starvation, sex or sleep deprivation, heat, mechanical stress, and electric foot shocks) generally increased WAFO, while positively valenced stimuli (social grouping, feeding, and relief from stress) decreased it (Lueningschroer-Wang et al., 2025). This group also found that both overexpression and downregulation of the serotonin transporter, as well as thermogenetic activation of NPF + neurons, decreased WAFO. Thermogenetic suppression of dopaminergic reward neurons increased WAFO. Finally, WAFO decreased significantly following the consumption of diazepam-containing food, consistent with previous results (Mohammad et al., 2016).

Hence, it is possible that flies could be used in the future as a neurogenetic model for emotion primitives and defensive behaviors, yet additional research is needed to validate this model. This could include adapting other tests of defensive behaviors in rodents, beyond the open field test, to flies. For instance, flies and mice could be tested on novelty-suppressed feeding (Bodnoff et al., 1988) or a food preference assay, to characterize similarities in other anxiety-like or anhedonia-like behaviors. Researchers could then apply the aforementioned manipulations of GABAergic and serotonergic signaling and measure changes across a battery of behaviors, to increase evidence supporting the use of flies as a neurogenetic model for human internalizing-like behaviors.

### Models for externalizing-like behaviors

In addition to studying *D. melanogaster* as a potential model for stress-induced internalizing-like behaviors, flies have also been used to study behaviors relevant to externalizing disorders. Again, we do not believe that flies have externalizing emotions in the same way as humans, but changes in internal states may stimulate behaviors, such as aggression and hyperactivity, that are analogous to human externalizing symptoms. It should also be clarified that aggression and activity are not disorders, but these phenotypes may present as the outward manifestations of neuronal disorders. For example, hyperactivity is strongly linked to externalizing psychopathology in humans as a key symptom of attention-deficit hyperactivity disorder (ADHD). ADHD patients with pronounced hyperactivity are more likely to demonstrate hallmark features of externalizing disorders, including impulsivity and aggression (Ahmad and Hinshaw, 2017). Aggression and locomotor activity are two behaviors that can be quantified easily across species. In *D. melanogaster*, not all hyperactivity may be reflective of externalizing-like behavior. However, in some cases, there may be a common molecular substrate between fly hyperactivity and the hyperactivity observed in human ADHD, suggested by findings such as loss-of-function mutations in the dopamine D1 receptor ortholog DopR exacerbating environmentally-stimulated hyperactivity (Lebestky et al., 2009).

### *Drosophila melanogaster* studies of aggression

As previously noted, social isolation has been found to enhance aggression in flies (Hoffmann, 1987; Hoffmann and Cacoyianni, 1989; Wang et al., 2008), similarly to food deprivation (Edmunds et al., 2021). A study examining genetic variance and gene–environment interactions in aggression in socially isolated and socially experienced individuals established that significant genetic variation exists in the aggressive response to social isolation (Rohde et al., 2017). The findings of this study support a polygenic basis of aggression in *D. melanogaster*, with several of these genes having orthologs previously implicated in mouse aggression and human neurological disorders. The *D. melanogaster* genes *cac*, *dysf, Fas2, Fas3, ftz-f1, kirre, loco,* and *rst* have orthologs in mice associated with aggression. *Dysf* and *Fas2* also have human orthologs associated with bipolar disorder (Psychiatric GWAS Consortium Bipolar Disorder Working Group, 2011) and suicide attempts in depression and bipolar disorder (Mullins et al., 2014), respectively, in mixed-sex patient populations. Multiple candidate genes previously implicated in both *D. melanogaster* aggression and human disorders emerged, including *Cad87A, CG42458, Dys, Gug, Ptp99A, Rbfox1, Ten-a,* and *Tl*. The human orthologs of these genes have been associated with bipolar disorder, anxiety, depression, schizophrenia, and Alzheimer’s disease.

Although the study above (Rohde et al., 2017) has been critiqued for lack of replicability (Chowdhury et al., 2021) and the use of a behavior test that may not truly capture aggression (Kravitz and Fernandez, 2015), other studies have identified neuromodulatory systems and genes implicated in fly aggressive behavior. Increasing serotonin pharmacologically and genetically in serotonergic circuits increases aggression in *D. melanogaster*, as does silencing neuropeptide F (NPF) circuitry (Dierick and Greenspan, 2007). Serotonin and neuropeptide Y, the vertebrate ortholog of NPF, have been associated with aggression (Karl et al., 2004; Popova, 2006) among other behaviors (Lucki, 1998; Pedrazzini et al., 2003; Carvajal et al., 2006). These two neuromodulatory systems may be evolutionarily conserved across species and oppose each other to regulate aggression. Additionally, neuropeptide signaling from neurosecretory cells within the *pars intercerebralis* (PI) is a major molecular regulator of aggression in *D. melanogaster* (Davis et al., 2014). The PI functions as the *Drosophila* equivalent of the mammalian hypothalamus, and individual knock down of the genes *tailless* and *Atrophin* in the PI increases fly aggression (Davis et al., 2014). The physical interaction of the protein products encoded by these genes in the PI may modulate aggressive behavior by controlling neuropeptide release.

### *Drosophila melanogaster* studies of locomotor activity

In addition to aggression, hyperactivity also follows social isolation in male and female flies (Ganguly-Fitzgerald et al., 2006; Li et al., 2021), which may be due to histone modifications altering the expression of activity-related transcription factors in dopaminergic neurons (Agrawal et al., 2019). In addition to isolation, short term variable stress (Ramos-Hryb et al., 2021) and starvation (Yu et al., 2016; Chi et al., 2021) have been shown to stimulate hyperactivity in male and female flies. Starvation stress may induce hyperactivity in flies to facilitate food location and acquisition. This behavior can be attributed to the binding of adipokinetic hormone, the insect analog of glucagon, to its receptor in adipokinetic hormone receptor+ octopaminergic neurons. Silencing these neurons, blocking octopaminergic transmission in these neurons, or binding *D. melanogaster* insulin-like peptides to the insulin-like receptor either eliminates or suppresses starvation-induced hyperactivity in females (Yu et al., 2016). It has also been found that a population of male-specific P1 neurons regulates sleep, feeding, and courtship in male *Drosophila* (Zhang et al., 2018). These cells could be important for understanding the neurochemical basis of how social isolation and starvation stress induce similar externalizing-like phenotypes (aggression and locomotor activity), as well as sex differences in the behavioral response to these stressors.

### Behavioral sex differences in fly models of stress

As noted in the foregoing, behavioral sex differences in internalizing and externalizing disorders may also manifest in *D. melanogaster* models of stress. It has been previously suggested that *D. melanogaster* behavioral responses to stress may depend on sex and sexual maturity, and that stress-induced changes in one behavioral test may not predict performance in others (Neckameyer and Nieto, 2015). It should be noted that sex determination is more strongly regulated by genetics in *D. melanogaster* compared to mammals, which rely on both genetics and sex hormones for sexual differentiation. The ratio of X chromosomes to autosomes determines sex in each cell of the fruit fly embryo through a cascade of pre-mRNA alternative splicing events, without the influence of sex hormones (Gilbert, 2000; Heller, 2010). Stress may uncover latent sex differences in fly behaviors, which are generated as a result of sexual identity-based differences in certain populations of cells.

One study examining the effects of isolation versus social contact on aging found that lifelong same-sex pairing decreased lifespan more in males than in females (Leech et al., 2017). While same-sex pairing in females protected against functional senescence, measured by climbing ability, same-sex pairing produced climbing deficiencies in males. The detrimental effect of same-sex pairing on lifespan doubled in males in combination with wounding. These results suggest that male and female *D. melanogaster* respond differently to social contact and isolation, and that the adverse effects of social contact on longevity in male–male pairings may be due to the stress of increased competition for mates.

Another report that studied sex differences in *D. melanogaster* behavioral responses to chronic stress found that stressed females showed more behavioral changes than stressed males, and that the effects on behavior varied based on whether the stress could be categorized as abiotic (mechanical perturbation) or biotic (adult crowding) (Lall et al., 2019). In this study, adult crowding produced more sex differences in behavior than mechanical stress in tests of motivation and locomotion (Lall et al., 2019). Following chronic overcrowding, females showed greater anhedonia-like behavior, measured through decreased interest in glycerol-feeding, while males showed less. Females also had a decreased tendency to explore a novel environment, and females’ activity declined while males became hyperactive. Different results followed chronic vibration stress. After experiencing chronic vibration, both males and females demonstrated increased anhedonic-like behavior and activity. The only sex difference observed following vibration was decreased exploratory behavior in females that was not evident in males.

Hence, flies may respond differently to stress based on whether it is social or non-social in nature. The multiple behavioral sex differences observed following overcrowding demonstrate how a common stressor may influence internal state differentially in females versus males to produce divergent phenotypes. These divergent behavioral responses to social stress could potentially relate to female versus male reproductive demands and drives. The perception of increased competition for food and mates in a crowded environment could drive males to eat more glycerol and increase their activity, to locate these resources faster. In female *Drosophila*, glycerol is metabolized to support oogenesis (Heier and Kühnlein, 2018). If population density is too high, females may divert resources away from reproduction and consequently be less interested in glycerol-feeding or less motivated by food overall (leading to lower locomotion).

### Molecular and neuromodulatory mechanisms in fly models of stress and stress-related behaviors

Well-characterized in mammalian systems, neurotransmitters, including the biogenic amines serotonin, dopamine, and octopamine, have been studied extensively as a mechanistic link between stress and behavioral changes in *D. melanogaster*. Stress can induce depression-like phenotypes in flies associated with the depletion of serotonin, dopamine, and octopamine, which can be rescued through several approaches with mixed success (Ries et al., 2017; Araujo et al., 2020; Ramos-Hryb et al., 2021).

### Serotonin

Serotonin, or 5-hydroxytryptamine (5-HT), signaling pathways are evolutionarily conserved across a wide range of species (Turlejski, 1996; Hay-Schmidt, 2000; Buznikov et al., 2001), resulting in many similarities in serotonin signaling between flies and mammals in the areas of behavioral regulation, mechanism of action, and conservation of receptor families. Serotonin is known to modulate locomotion, sleep and circadian activity, feeding, aggression, anxiety and depressive-like behavior, and learning and memory in *D. melanogaster* (Bacqué-Cazenave et al., 2020). Serotonin also regulates many of these behaviors in mammals, including sleep–wake states (Monti, 2011), feeding (Vickers and Dourish, 2004; Voigt and Fink, 2015; D’Agostino et al., 2018), aggression (Miczek et al., 2001; Howell et al., 2007), anxiety-like behavior (Barnes and Sharp, 1999), depressive-like behavior (Artigas, 2013), and learning and memory (Buhot, 1997; Pittenger and Kandel, 2003). In both flies and mammals, serotonin exerts its effects through interactions with G protein-coupled receptors, which share considerable sequence similarity between species. *D. melanogaster* has the receptors 5-HT1A, 5-HT1B, 5HT-2, and 5HT-7 (Witz et al., 1990; Saudou et al., 1992; Colas et al., 1995), which are functional orthologs of the 5-HT1A, 5-HT2, and 5-HT7 receptors in mammals. However, despite these similarities, serotonin signaling differs between species due to mammals’ greater numbers of serotonergic neurons, diversity and number of receptor families, and complexity of neural circuits (Kasture et al., 2018). Although having a smaller and less complex brain can limit the extent to which *D. melanogaster* can model human disorders, the relative simplicity of the fly brain also allows for a reductionist study of the effects of neuromodulatory signaling on behavior.

Serotonin binding to its receptors influences the activity of cAMP-protein kinase A (PKA) (Ganguly et al., 2020), PLC-IP3 (Blenau et al., 2017; Tierney, 2018), GABAergic, and cholinergic signaling pathways (Alekseyenko et al., 2019) in *D. melanogaster*. Alterations in cAMP-PKA and PLC-IP3 signaling can induce downstream changes in gene expression, synaptic plasticity, and neuronal excitability. Serotonin also modulates the activity of GABAergic and cholinergic neurons, which, respectively, reduce and elevate aggressive behavior in males (Alekseyenko et al., 2019).

The literature on serotonergic signaling and aggression in *D. melanogaster* is substantial. Treatment with serotonin precursor 5-HTP or activation of 5-HT neurons enhances male aggression, while serotonergic neuronal silencing tends to decrease male aggression (Dierick and Greenspan, 2007; Alekseyenko et al., 2014; Hu et al., 2020). However, the effects of serotonin on aggression may be receptor subtype-specific. Although one report found that activation of the 5-HT1A receptor induces aggression in socially isolated male flies (Johnson et al., 2009), another group found that activating 5-HT1A neurons decreases male aggression (Alekseyenko et al., 2014). This discrepancy may be reconciled by more recent research, which has identified two distinct 5-HT1A receptor-expressing neuron types with opposing functions in regulating aggression (Alekseyenko et al., 2019). Activation of the first type, inhibitory GABAergic neurons, results in decreased aggression. Activation of the second type, excitatory cholinergic neurons, increases aggression. It is hypothesized that these two cell types converge in a common sensory integration area, the LC12 optic glomerulus, and that downstream signaling pathways from this brain region may refine the aggression response (Alekseyenko et al., 2019). The activation of specific receptor subtypes may also produce different forms of aggressive behaviors. For example, activation of 5-HT2 receptors has been associated with lunging and boxing, but manipulation of the 5-HT1A receptor alters wing threats and fencing (Johnson et al., 2009).

### Dopamine

Dopaminergic signaling is another neuromodulatory system with functional parallels between flies and mammals. In both *Drosophila* and mammals, dopamine has been found to mediate locomotor activity, sexual behavior, response to drugs of abuse, memory, sleep, circadian rhythms, and aggression (Wilson et al., 1991; Mani et al., 1994; Yellman et al., 1997; Missale et al., 1998; Neckameyer, 1998; Andretic et al., 1999; Bainton et al., 2000; Schwaerzel et al., 2003; Willuhn et al., 2010; Riemensperger et al., 2011; Alekseyenko et al., 2013; Berry et al., 2015; Yamagata et al., 2015; Korshunov et al., 2017; Duszkiewicz et al., 2019; Hasegawa et al., 2022; Dai et al., 2025). In mammals, dopamine acts as a primary neurotransmitter in mesolimbic, mesocortical, nigrostriatal, and tuberoinfundibular pathways to modulate reward, motivation, motor control, and hormonal control (Björklund and Dunnett, 2007; Muthuraman et al., 2018; Tian et al., 2022). In *D. melanogaster*, clusters of dopaminergic neurons in the brain and ventral nerve cord project their axons to the mushroom bodies, central complex, and ventral lateral protocerebrum to regulate memory, locomotion, and aggression (White et al., 2010; Alekseyenko et al., 2013; Marquis and Wilson, 2022). Beyond the overlap in dopamine-regulated behaviors, the majority of genes involved in dopamine synthesis, secretion, and signaling are also conserved across species (Yamamoto and Seto, 2014). Several receptors and transporters involved in signal reception within dopaminergic pathways and dopamine reuptake, respectively, are conserved. *Drosophila* have four G protein-coupled dopamine receptors: *DopR* (Gotzes et al., 1994; Sugamori et al., 1995) and *DopR2* (Feng et al., 1996; Han et al., 1996), which are orthologs of mammalian D1 receptors; *D2R* (Hearn et al., 2002), which is a D2-like receptor; and *DopEcR* (Srivastava et al., 2005), a non-canonical receptor.

However, there is some evolutionary divergence from mammals in genes involved in dopamine metabolism. Flies either (a) recycle dopamine through glial reuptake, whereby dopamine undergoes *β*-alanylation (Hovemann et al., 1998), and the resulting product is converted back to dopamine (True et al., 2005), or (b) metabolize dopamine via N-acetylation (Hintermann et al., 1996; Paxon et al., 2005). These processes differ from the mammalian metabolic mechanisms of oxidation and methylation (Meiser et al., 2013). Additionally, there are differences between flies and mammals regarding the organization of dopaminergic neurons and dopaminergic receptor subtypes. Dopaminergic neurons are largely concentrated in the substantia nigra and ventral tegmental area of the midbrain in mammals (Hegarty et al., 2013). In flies, they are found in specific clusters throughout the brain, such as clusters PAM, PPL, and PPM (Mao and Davis, 2009). The mammalian brain also has a greater number of dopaminergic receptor subtypes with greater diversity of splice variants and polymorphisms compared to the fly brain (Jackson and Westlind-Danielsson, 1994; Missale et al., 1998; Seeman et al., 2000; Hearn et al., 2002; Gurevich et al., 2016; Karam et al., 2019; Magistrelli et al., 2021), giving rise to a larger and more complex dopaminergic system.

There is substantial evidence that dopamine signaling in *D. melanogaster* is associated with behavioral and physiological indicators of stress. Dopaminergic transmission increases heart rate (Johnson et al., 1997), modulates arousal (Andretic et al., 2005), and increases with heat stress (Gruntenko et al., 2004). At baseline, adult female *D. melanogaster* also have higher levels of dopamine than adult males (Denno et al., 2015). Dopamine has been found to play a role in female sexual receptivity (Neckameyer, 1998) and to modulate the effects of drugs of abuse, including cocaine, nicotine, and ethanol (Neckameyer, 1998; Bainton et al., 2000). Stressors such as starvation, oxidative stress, and mechanical stress have been found to disrupt female sexual receptivity and ovarian development, potentially through alterations in tyrosine hydroxylase activity, which plays a critical role in dopamine biosynthesis (Neckameyer and Weinstein, 2005).

Social space among flies is also modulated by dopaminergic signaling, with dose-dependent effects differing by sex. This finding suggests a role for sexually dimorphic dopaminergic neurons in maintaining appropriate social spacing (Fernandez et al., 2017). Previously socially isolated male and female flies show reductions in physical distance to other flies, while prior mating increases social distance (Simon et al., 2012). Socially isolated male and female flies also decrease their sleep and have roughly one-third the level of whole brain dopamine as their socially enriched, longer-sleeping siblings (Ganguly-Fitzgerald et al., 2006). Both silencing dopaminergic circuitry and increasing endogenous dopamine prevent social deprivation from altering sleep, suggesting that dopaminergic transmission is crucial to this form of experience-dependent behavioral plasticity (Ganguly-Fitzgerald et al., 2006). An additional study found that social isolation-induced dopamine depletion, as well as dopamine recovery to normal levels following 3 days of group-housing, was specific to male flies (Yost et al., 2024). The previously mentioned study (Ganguly-Fitzgerald et al., 2006) found reductions in whole head dopamine levels in mixed-sex isolated versus socially enriched control flies. It is possible that this result was driven by male-specific dopamine depletion, given that this research group did not perform sex-specific comparisons, but further investigation is needed to explore whether these studies support or contradict one another.

Lastly, differential sensitivity of dopaminergic receptors or differences in receptor subtype expression may account for sex differences in behaviors, such as locomotion (Yellman et al., 1997). Dopaminergic neurons may also be differentially vulnerable to neurodegeneration on the basis of sex. Female *D. melanogaster* have elevated expression of vesicular glutamate transporter in dopaminergic neurons relative to males (Buck et al., 2021). This sex difference is conserved across flies, rodents, and humans and may confer protection to females against aging-related neurodegeneration and locomotor deficits (Buck et al., 2021).

### Octopamine

Octopamine, the insect structural analog of mammalian norepinephrine, is expressed in roughly 70–100 neurons in the *D. melanogaster* nervous system and modulates a range of behaviors, from ovulation to learning (Monastirioti, 2003; Schwaerzel et al., 2003). Neural octopamine is required for *D. melanogaster* aggression (Zhou et al., 2008). Silencing of octopaminergic neurons and genetic knockdown or mutant approaches have generally yielded decreases in male and female aggressive behavior (Hoyer et al., 2008; Zhou et al., 2008; Watanabe et al., 2017; Sherer et al., 2020), while activation of octopaminergic neurons generates male aggression (Watanabe et al., 2017; Jia Y. et al., 2021). In one of these studies, the release of both octopamine and glutamate from neurons expressing both neurotransmitters was found to be necessary for male aggression (Sherer et al., 2020). Another research group induced a depression-like state in male flies, characterized by reduced climbing motivation, using a battery of chronic stressors, including vibration, overcrowding, and food deprivation. It was found that the depression-like state could be relieved through consumption of 5-HTP, fluoxetine, or sucrose, all of which increase serotonin in the brain (Hermanns et al., 2022). A sugar-induced restoration of climbing motivation was associated with octopaminergic signaling from the ventral unpaired medial neurons in the subesophageal ganglion to dopaminergic protocerebral anterior medial (PAM) neurons. The dopaminergic PAM neurons and serotonergic dorsal paired medial (DPM) neurons both synapse with cells in the mushroom bodies to modulate and relieve the stress-induced depression state. These findings reiterate the important ways in which monoaminergic signaling systems work together in behavioral regulation, and how disruption of these systems by stress dysregulates behavior.

In the context of stress, octopamine has also been identified as a substrate for starvation-induced foraging behavior, but not feeding itself, in virgin female flies (Yang et al., 2015). In males, overexpression of tyramine *β* hydroxylase (TβH), an enzyme involved in octopamine synthesis, increases aggression in group-housed flies but not socially isolated flies (Zhou et al., 2008). This result could be attributed to either a ceiling effect in the already high levels of aggression demonstrated by isolated flies, or a role for octopamine in the social regulation of aggression. Octopamine may also modulate male aggression by inducing the production and secretion of Dsk, a satiety-related neuropeptide that will be discussed in the subsequent section (Williams et al., 2014). Interestingly, male flies have been found to express higher levels of mRNA for the octopaminergic receptor Octα2R than females, which could be attributed to higher numbers of Octα2R + neurons in males or male-specific upregulation of *Octα2R* expression (Qi et al., 2017). It is possible that sex differences in gene expression related to octopaminergic signaling could contribute to the generation of sex-specific behaviors, such as male aggression.

### Drosulfakinin

Neuropeptides are another subset of neuromodulators that have been found to play a role in behavior change driven by social stress. Cholecystokinin (CCK) is a mammalian gut hormone that also functions as a neurotransmitter. CCK + neurons regulate aggression, anxiety, and social defeat responses in rodents (Vasar et al., 1993; Panksepp et al., 2004; Becker et al., 2007; Li et al., 2007), and CCK-4 has been shown to induce panic attacks in humans (Bradwejn et al., 1990; Tõru et al., 2010). Dsk, the fly ortholog of CCK, has been implicated in regulating gut function, feeding and satiety, hyperactivity, aggression, locomotor behavior, and synaptic plasticity during the development of the neuromuscular junction (Nässel and Williams, 2014). The *D. melanogaster* genome encodes two distinct receptors to which Dsk binds: CCKLR1 (CCKLR-17D1) and CCKLR2 (CCKLR-17D3). It has been shown recently that the binding of Dsk to CCKLR-17D1 in insulin-producing cells modulates selectivity in mating choice in males (“male choosiness”), and that broad activation of Dsk + neurons rescues the adverse effect of female pheromone exposure on male lifespan (Fedina et al., 2023). Dsk signaling in male flies has also been implicated in early life social memory, which may have lasting effects on social behavioral plasticity (Jeong et al., 2024). Both *Dsk* and *CCKLR-17D1* mutants show deficits in larval stress-induced escape behavior (Chen et al., 2012), and knockdown of these genes in the PI in adults increases aggression in socially isolated males (Agrawal et al., 2020). Consistent with the fact that social isolation increases aggression in *D. melanogaster*, another group has found that chronic social isolation decreases *Dsk* expression in males, which correlates with decreased sleep and increased feeding behavior (Li et al., 2021). Although there is abundant evidence that Dsk signaling may play a role in stress-related phenotypes and social behaviors in *D. melanogaster*, little is known about whether chronic stress induces sex differences at the transcriptomic level in this pathway, and if such differences could alter behaviors such as activity, feeding, or aggression.

### Neuropeptide F

Neuropeptide F (NPF), the fly homolog of mammalian neuropeptide Y (NPY), has also been linked to essential behaviors which are impacted by stress. In mammals, NPY is involved in the regulation of diverse biological functions, including energy balance, circadian rhythms, feeding, reproduction, anxiety, learning and memory, and alcohol dependence (Lee et al., 2006). There is also some evidence that NPY pathways mediate physiological and behavioral stress responses differently between males and females (Painsipp et al., 2011; Forbes et al., 2012). In *D. melanogaster*, NPF regulates feeding, wakefulness, metabolism, and reproduction (Nässel and Wegener, 2011; Chung et al., 2017). A related class, short NPFs, which belong to a separate invertebrate neuropeptide family from NPFs but bind to NPY-like receptors, have been associated with feeding and growth, stress response, movement, olfaction, hormone release, and learning and memory (Nässel and Wegener, 2011). One study has found that inhibitory signaling from NPF + neurons to dopaminergic neurons confers resilience to chronic stress-induced learning deficits through the maintenance of normal autophagic flux (Chen et al., 2023). Silencing NPF + cells has also been shown to increase aggression in socially isolated males (Dierick and Greenspan, 2007) and decrease male courtship behavior in group-housed controls (Lee et al., 2006). *NPF* also demonstrates sex-specific expression patterns in *D. melanogaster*, which may influence sexual dimorphism and sex differences in behavior. Male flies have neurons which express male-specific NPF under regulation of the transformer (tra) sex determination pathway and may modulate male courtship (Lee et al., 2006). Male-specific *NPF* expression in neurons may also be regulated by circadian factors, suggesting a role of these cells in generating sex-specific behaviors which are also clock-controlled (Lee et al., 2006). There is currently limited work examining whether NPF signaling and sex-nonspecific behaviors diverge under stress between males and females, providing a needed avenue for future research.

### Corazonin

Finally, corazonin (Crz) is a lesser studied but highly conserved neuropeptide in insects that may contribute to sexually dimorphic neural circuitry and behavioral responses to stress. *D. melanogaster* cells expressing *Crz* have receptors for two diuretic hormones, DH44 and DH31 (Johnson et al., 2005), which are related to the mammalian stress hormones corticotropin releasing factor and calcitonin gene-related peptide (Bale and Vale, 2004; Taylor and Samson, 2005). It has been suggested that Crz plays important functions in development and survival by regulating growth, feeding, mating behavior, and ethanol sensitivity (Khan et al., 2021). Although Crz is expressed in dorsomedial, dorsolateral, and ventral nerve cord neurons in the fly nervous system, there is a small population of four neurons in the abdominal ganglion which are present only in males and are involved in ejaculation and copulation duration (Lee et al., 2008). A study examining the role of *Crz*-expressing neurons in stress sensitivity and stress-related behavior identified sex differences in lifespan following starvation, osmotic, or oxidative stress. Activation of Crz + neurons decreased lifespan only in males, despite neural silencing improving lifespan in *both* sexes under conditions of starvation or oxidative stress (Zhao et al., 2010). This study also found male-specific effects of Crz + neuron activation and silencing on motor activity, with silencing resulting in male hyperactivity and activation resulting in male hypoactivity relative to wild type flies. Starvation and osmotic stress decreased transcript expression of *Crz* more in males compared to females, and males with silenced Crz + neurons showed elevations in dopamine in the hemolymph relative to parental genotypes. Activation and silencing of corazonin neurons did not alter dopamine levels compared to parental genotypes in females (Zhao et al., 2010). Finally, a separate sex-reversal study found that feminizing Crz + neurons in male flies was sufficient to reduce time to sedation by ethanol to female-like levels (Oyeyinka et al., 2022). Taken together, the results of these experiments suggest that corazonin neurons may receive sex-specific inputs or may directly or indirectly signal to sex-specific downstream targets to produce sex differences in stress phenotypes. To date, no studies have explored the effects of social isolation on corazonin neural signaling and behavior in *D. melanogaster*.

### Molecular and cellular sex differences in social isolation stress

Overall, the studies outlined below offer many potential genetic targets for exploring male- versus female-specific cell types and circuits underlying stress-induced and social context-dependent sex differences in behaviors (Table 3). In the context of social isolation, a recent review (Yadav et al., 2024) summarized extensive research on the genes, neuromodulators, cell types, and behaviors that are differentially altered by single versus group housing conditions, with courtship, sleep, and aggression being the most widely studied behaviors. At a circuit level, both sexually monomorphic and dimorphic cell types underlie the *Drosophila* aggression response (Chiu et al., 2021). Common aggression-promoting (CAP) neurons mediate the approach (appetitive phase) of aggression in both sexes, but the attack (consummatory) phase relies on male-specific aggression-promoting (MAP) neurons in males and fpC1 neurons in females (Chiu et al., 2021). Social isolation may enhance aggressive behavior in both sexes by strengthening these monomorphic-to-dimorphic functional connections.

**Table 3:** Genes implicated in social experience-dependent and stress-induced phenotypic changes.

Gene	Manipulation	Sex	Social experience	Phenotypes	References
*Dsk*	Genetic knockdown Chronic social isolation ↓ expression	Males Males	Social Isolation Social Isolation	Aggression ↑ Sleep ↓, feeding ↑ (correlative)	Agrawal et al. (2020) Li et al. (2021)
*Obp69a*	Social isolation ↑ expression Group housing with males, but not females, ↑ expression	Males Females	Social Isolation Group Housing	Aggression ↑ Sexual receptivity ↑	Bentzur et al. (2018)
*Cyp6a20*	Social enrichment ↑ expression (relative to isolation)	Males	Group Housing	May contribute to aggression ↓ in combination with *Obp69a* expression ↓	Bentzur et al. (2018)
*NPF*	Neural silencing Neural silencing	Males Males	Social Isolation Group Housing	Aggression ↑ Courtship ↓	Dierick and Greenspan (2007) and Lee et al. (2006)
*Tk*	Social enrichment ↓ expression (relative to isolation)	Males	Group Housing	Spontaneous locomotion ↓	Zhao et al. (2024)
*DH44*	Social enrichment ↑ expression (relative to isolation)	Males	Group Housing	Spontaneous locomotion ↓	Zhao et al. (2024)
*Crz*	Neural activation Neural silencing	Males Females Males Females	Group Housing Group Housing Group Housing Group Housing	Lifespan ↓ under starvation, osmotic, or oxidative stress; activity ↓; no change in dopamine in hemolymph Lifespan ↑ under starvation or oxidative stress; no change in activity; no change in dopamine in hemolymph Lifespan ↑ under starvation, osmotic, or oxidative stress; activity ↑; dopamine in hemolymph ↑ Lifespan ↑ under starvation or oxidative stress; no change in activity; no change in dopamine in hemolymph	Zhao et al. (2010)

In addition to *Dsk* downregulation (Agrawal et al., 2020), social isolation-induced aggression has also been linked to upregulation of *Obp69a* and downregulation of *Cyp6a20* (Bentzur et al., 2018). Interestingly, *Obp69a*, a gene encoding an odorant binding protein, and *Cyp6a20*, a gene encoding a cytochrome P450, are expressed in a sexually dimorphic manner. *Obp69a* is naturally overexpressed in males relative to females and increases only in males following single housing. In females, expression of *Obp69a* does not seem to be affected by single or group housing with same-sex conspecifics, but it increases when they are group-housed with males. Expression of *Cyp6a20* does not vary based on social environment in females but is significantly reduced in males following single housing (Bentzur et al., 2018). The authors believe that the inverse regulation of *Obp69a* by social environment in males versus females may promote mating, by decreasing aggression and increasing aggregation in groups for males and enhancing sexual receptivity for females. It is plausible that pheromone-sensing pathways may be altered by social context differentially in males and females, due to male-specific initiation of courtship behavior. This is supported by studies cited earlier in this review, which found social experience to enhance Or47b neural activity and stimulate courtship in mature adult males (Sethi et al., 2019; Zhao et al., 2020).

NPF, which is altered by stress and associated with stress-induced behavior change, is regulated in both a sex-specific and sex-nonspecific manner (Lee et al., 2006). Male-specific *NPF* expression is regulated by fruitless (fru), a transcription factor which plays a critical role in the sex determination hierarchy in the fly central nervous system and is required for normal male courtship. *NPF* is also regulated by clock factors in a subset of male dorsolateral neurons, suggesting that this gene may play a role in sexually dimorphic courtship behavior which is also circadian (Lee et al., 2006). Since NPF + neuronal silencing increases aggression in socially isolated males and decreases courtship in group-housed males, it would be interesting to explore whether social isolation impacts *NPF* gene expression in a sex-specific way (e.g., decreases *NPF* expression in males), and, if so, whether *NPF* expression changes in this particular population of neurons. P2 neurons expressing *NPF* have also been identified as the cells through which chronic social isolation reduces sleep and increases feeding, but this study only tested male flies (Li et al., 2021).

In addition to NPF, tachykinin (Tk) is a neuropeptide which may play sex-specific roles in stress-related behaviors. Substance P, a member of the Tk family, has been linked to the initiation of aggressive behavior in mammals (Katsouni et al., 2009) and may perform a similar function in insects. A class of neurons in the male fly brain that express the male-specific form of *fru* and release *D. melanogaster* Tk have been found to generate male-specific aggression (Asahina et al., 2014). A recent report has also found that in male flies, social enrichment (relative to social isolation) upregulates *Diuretic Hormone 44* (*DH44*) and downregulates *Tk* to decrease spontaneous locomotor activity (Zhao et al., 2024). These authors also uncovered a neural circuit in which male-specific P1 neurons stimulate movement through positive feedback with interneurons expressing both *doublesex* (*dsx*) and *Tk* and inhibit movement with negative feedback from interneurons co-expressing *dsx* and *DH44* (Zhao et al., 2024). This neural circuit could be integral to understanding how changes to the social environment produce sexually dimorphic changes in innate behaviors. Taken together, the results of these studies allude to male-specific neural substrates in flies for two externalizing behaviors, aggression and hyperactivity.

Interestingly, the feminization of cholinergic neurons in male flies via expression of *tra* also increases aggression (Mundiyanapurath et al., 2009). *Tra* is an important gene in the sex determination hierarchy that is only functional in females. It has been found that a small number of cholinergic cells in female fly brains, pC1 dsx + neurons, are required for female hyperaggression (Palavicino-Maggio et al., 2019). Although females are typically less aggressive than males at baseline (Kravitz and Fernandez, 2015), they will become more aggressive, similarly to males, following social isolation (Palavicino-Maggio and Sengupta, 2022). Further research is needed to determine if social isolation induces female aggression by altering gene expression in such a way that makes pC1 dsx + or other cholinergic neurons more active.

### Molecular and cellular sex differences in crowding stress

In *D. melanogaster*, there has been more research conducted on larval than adult crowding. Larval crowding has been associated with transcriptomic changes in adulthood, including the upregulation of genes relating to metabolism and DNA replication and downregulation of genes involved in immune signaling. Downregulation was also observed in gene classes relating to excretion and recycling pathways, namely taurine metabolism and lysosome and ABC transporters (Morimoto et al., 2023). Overall, high density living early in life may remodel metabolic pathways in flies to cope with a limited-resource environment and build tolerance to toxic compounds.

However, one study has suggested that the stress resistance built in adulthood by larval crowding may not be the same in both sexes. During adult heat stress, it was found that after multiple generations of larval crowding, males but not females improved their heat shock tolerance (Kapila et al., 2021). Crowding increased expression of the heat shock protein *Hsp70* in larvae, but *Hsp70* expression in adulthood did not account for the observed sex difference in heat shock tolerance, as there were no *Hsp70* expression differences in the adult populations. Thus, the molecular basis of this observed sex difference in heat stress tolerance remains largely speculative. Another study of larval crowding in the fruit fly *Bactrocera tryoni* found that high density early living decreased emergence, body weight, energetic reserves, flight ability, and fertility and increased feeding in adulthood (Morimoto et al., 2019). Although flies of both sexes from the larval crowding treatment increased their total food consumption (relative to body weight), males ate more yeast, while females ate more sucrose compared to their respective uncrowded controls. Further research is needed to uncover the molecular mechanisms by which larval crowding induces sex differences in stress sensitivity and nutrient preference in adulthood.

In adult flies, one study (Chen et al., 2023) used adult crowding stress, in combination with mechanical shock, to examine the molecular and neuronal mechanisms underlying chronic stress-induced learning deficits. Learning and memory impairment is a common behavioral symptom of depression in humans and other mammals. Four days of crowding and mechanical shock treatment were sufficient to induce learning deficits (Jia J. et al., 2021), which correlated with disrupted autophagic degradation in neurons. RNA-seq differential expression analysis from fly heads collected after 96 h of stress treatment revealed changes in gene expression in the autophagy-lysosome pathway. This is an evolutionarily conserved stress pathway (Kroemer et al., 2010) that degrades abnormal intracellular macromolecules to maintain cellular homeostasis. This pathway has also been implicated in both neurodegenerative diseases (Nixon, 2013) and depression (Jia and Le, 2015; Gassen and Rein, 2019). Activation of NPF + cells during chronic stress protects against learning deficits by inhibiting dopaminergic neurons and thus maintaining autophagic flux (Chen et al., 2023). Future studies should examine whether these specific molecular changes in the autophagy-lysosome pathway can be linked to depressive-like behavior or other stress responses in flies.

Lastly, one paper studying adult restraint stress, which bears some similarity to overcrowding in that the flies were restricted in physical space in a group, found that restraint of fly fathers induced gene expression changes in the offspring (Seong et al., 2020). It was found that paternal restraint disrupted heterochromatin, upregulated the expression of genes involved in one-carbon metabolism, and downregulated the expression of genes involved in glycolysis and the tricarboxylic acid (TCA) cycle in F1 males. These authors identified a novel pathway by which paternal restraint stress reduces energy metabolism activity and produces epigenetic changes in male offspring to transmit stress information from somatic to germline cells. Overall, adult crowding or restraint stress seems to alter gene expression in *D. melanogaster* in a manner that is behaviorally maladaptive and detrimental to survival. However, the extent to which adult crowding or restraint stress produces behavioral sex differences in *D. melanogaster* remains fairly unknown, as well as the underlying molecular mechanisms if sexual dimorphism exists.

## Conclusion

Despite the *Drosophila* nervous system being smaller and simpler than that of vertebrates, genetic homology and conserved neural circuits between flies and mammals could be key to understanding latent sex differences by which stress differentially alters emotion primitives and behaviors. This knowledge is important for informing targets in the treatment of human disorders. This review has provided an extensive, but not exhaustive, overview of the molecular and systems level mechanisms through which flies generate sexually dimorphic, stress-induced behavioral responses.

Although there is overlap in the symptoms of human internalizing disorders and similar, stress-induced behaviors in flies, ongoing research is working to define the neurobiological mechanisms underlying these behaviors in flies and whether they have translational relevance to vertebrates. It is possible that orthologous genes could be identified in mammals whose roles have not yet been defined in stress-related circuits and behaviors. Another avenue for future research is to determine whether similar or different molecular pathways contribute to common phenotypes (e.g., sweet-seeking, activity) induced by different forms of stress (e.g., social isolation versus overcrowding) in flies. Additionally, many stress studies in *D. melanogaster* fail to explore the long-term consequences of stress exposure. For how long do social isolation and other forms of chronic stress exacerbate internalizing-like and externalizing-like behaviors? Do different behaviors emerge over time? Do sex differences in stress-induced molecular mechanisms and behaviors persist or evolve over time? These unanswered questions could provide further insight as to whether stress responses in flies resemble those observed in vertebrate animal studies.

Overall, *D. melanogaster* has long served as a useful model system for identifying conserved molecular pathways underlying fundamental behaviors and diseases. The genetic toolkit combined with established methods for stressing flies and quantifying internalizing-like and externalizing-like behaviors make *D. melanogaster* an attractive organism for exploring the molecular basis of sex differences in stress-induced behavior change. It is possible that evolutionarily conserved molecular mechanisms could play a role in human psychiatric and behavioral disorders that are precipitated by stress and manifest differently in males and females. Combined with knowledge from rodent models, increased understanding of these relationships could inform novel therapeutic interventions for stress-related disorders and emphasize the need for equal representation of both sexes in preclinical research.
